# Prudent care of head trauma in the elderly: a case report

**DOI:** 10.1186/1752-1947-8-448

**Published:** 2014-12-20

**Authors:** Allen GP Ross

**Affiliations:** Griffith Health Institute, Griffith University, Gold Coast Campus, Gold Coast, Australia

**Keywords:** Elderly, Health disparities, Health policy, Traumatic brain injury

## Abstract

**Introduction:**

Severe traumatic brain injury is a major public health problem that accounts for one-third of all deaths due to trauma in the United States. This case report illustrates some of the challenges faced by the elderly in accessing essential emergency services for traumatic brain injury.

**Case presentation:**

A 74-year-old Caucasian man presented with head trauma at his local acute care hospital (level III/IV) in Canada at 2:30 PM. He was triaged at 4:00 PM and was seen by the emergency room physician at 4:50 PM. His vital signs were normal, and his Glasgow Coma Scale score was 15/15 upon admission. A computed tomography–based diagnosis of acute subdural hematoma was subsequently made by a radiologist at 5:00 PM. A neurosurgical transfer was requested to the nearby tertiary trauma center (level I/II), but was initially refused by the neurosurgical resident on call. The patient’s condition slowly deteriorated until he became unconscious at 7:45 PM. The patient was intubated and transferred to the neurosurgical unit at 8:34 PM. He was seen by a consultant neurosurgeon at 9:30 PM, but surgery (craniotomy) was deemed not viable, given the patient’s age and the fact that his pupils were now fixed and dilated (Glasgow Coma Scale score 3/15). The patient was taken off life support at 1:00 AM the following morning and died shortly thereafter. The patient’s family made a formal complaint, but the decision by an independent medical review panel was that “the patient’s care was prudent, timely and professional.”

**Conclusions:**

Geriatric patients with severe head injury are less likely than their younger counterparts to be transferred to neurosurgical trauma centers. Protocol-driven care of the elderly can reduce mortality due to head trauma through the application of the Brain Trauma Foundation guidelines.

## Introduction

Globally, trauma ranks as the fourth leading cause of death overall and the leading cause of death among persons younger than 40 years of age [1, 2]. Each year more than 100 million people sustain traumatic injuries, resulting in close to 6 million premature deaths [1, 2]. Currently in Canada, there is an annual incidence of 200,000 patients hospitalized due to acute injury, with associated direct and indirect costs to the health-care system of close to $20 billion [2, 3].

Severe traumatic brain injury (TBI) is a major public health problem that accounts for one-third of all deaths due to trauma in the United States [4]. TBI costs the Canadian government approximately $7 billion per year in terms of health-care expenditures [4]. Protocol-driven care can reduce mortality and costs due to severe TBI through the application of the Brain Trauma Foundation guidelines [4, 5]. However, only two-thirds of Canadian patients ever reach a level I/II trauma center to receive definitive care [4].

Despite the Canada Health Act [6], which guarantees universal access to health care for all Canadians, significant challenges exist in delivering equitable access to trauma services across the country [3]. This case report illustrates some of the challenges faced in delivering trauma care, particularly to the elderly.

## Case presentation

A 74-year-old retired Caucasian man sustained a large contusion at the base of his skull as a result of a fall at home. He presented with head trauma at 2:30 PM at his local acute care hospital in Canada (level III/IV trauma center serving a catchment population of approximately 120,000 people). The patient was taking several medications, including rosuvastatin calcium for cholesterol, hydrochlorothiazide for blood pressure and dual anti-platelet therapy with aspirin and clopidogrel for previous transient ischemic attacks.

The patient was triaged at 4:00 PM, and a neck collar was applied. He was moved to an emergency room bed at 4:29 PM. At 4:50 PM, he was seen by the emergency room physician. Upon admission, he was alert and responsive, and his vital signs (body temperature, 35.5°C; heart rate, 77 beats/min; respiratory rate, 20 breaths/min; blood pressure, 149/96 mmHg; oxygen saturation, 95%) were stable. A neurological assessment revealed a Glasgow Coma Scale (GCS) score of 15/15. His pupils were equal, round and reactive to light. In his extremities, strength, reflexes and sensation were normal and symmetrical. He had no pronator drift, no abnormal cerebellar signs and no abnormal Babinski reflex.

Given the mechanism of his injury, history of amnesia, emesis and clopidogrel use, a computed tomographic (CT) scan of the patient’s head was ordered at 4:55 PM. A large, subcutaneous hematoma extending from the posterior parietal occipital region to the upper neck was noted. He had a right-sided subdural hematoma extending from the occipital through to the frontal lobe and superoinferiorly from the convexity down to the temporal lobe region. He had an acute hemorrhage along the falx and tentorium and a crescenteric focus of hemorrhage in the left frontal and anterior parietal region extending inferiorly to the temporal region. Minimal effacement of the sulci and lateral ventricle on the right side were noted. His basal cisterns were maintained, and no evidence of blood was found in the suprasellar or quadrigeminal plate cistern. No definite parenchymal hematoma was identified. The radiologist made a diagnosis of acute subdural hematoma on the basis of the CT scan at 5:00 PM. The attending physician requested an immediate transfer to the neurosurgical service at the nearby tertiary trauma center (level I/II), situated 30 minutes away. The neurosurgical resident on call at the trauma center initially refused the transfer until he had time to review the CT images. The patient’s condition slowly deteriorated while awaiting treatment, and he became unconscious at 7:45 PM that evening. He was unresponsive, his pupils did not react to light and he had no response to pain. He was given mannitol to his lower intracerebral pressure, and he was intubated. He was approved for transfer at 8:34 PM. The patient was seen by the consultant neurosurgeon at 9:30 PM, but surgery (craniotomy) was deemed not viable, given the patient’s age and the fact that his pupils were now fixed and dilated (GCS score 3/15).

The patient was taken off life support at 1:00 AM the next morning, and he died approximately 15 minutes later. The autopsy report from the medical examiner stated that the patient’s cause of death was trauma as a result of a fall at home. The neuropathological findings were as follows: subdural hemorrhage, predominately right-sided with marked right-to-left midline shift; subfalcine and transtentorial herniation; intracerebral white matter hemorrhage, bilateral; and Duret (secondary) hemorrhages, extensive, upper brainstem, diencephalic and pons.

The patient’s family made a formal complaint to an independent medical review panel. The panel’s decision was that “[t]he patient’s care was prudent, timely and professional.” The family was told that there was no process for them to appeal.

## Discussion

In the field of trauma, it is well known that the first hour after injury is the golden hour and that approximately 60% more lives can be saved if a patient is treated within that hour rather than later [7]. The elderly patient described in the present report waited with head trauma for 90 minutes before being triaged, and it took another 50 minutes until he was seen by an attending physician. Clearly, a “golden” opportunity to save his life was lost.

In Canada, as in many other countries in the developed world, the proportion of individuals 65 years of age and older is increasing. In Canada, the proportion of the elderly has doubled in the past 50 years, and they now number more than 5 million [7]. Research has shown that aggressive treatment of the elderly with survivable injuries results in the majority of them returning home, and 85% of these patients return to functional independence [7]. Moreover, the American College of Surgeons Committee on Trauma recommends that all trauma patients 65 years of age and older be considered for direct transport to a level I/II trauma center, regardless of injury severity [7, 8]. Despite these guidelines, however, evidence has shown that the undertriage of elderly patients to trauma centers is widespread [7], which is typified by the case of the patient reported here.

## Conclusions

Health disparities occur in wealthy nations, which impacts on the provision of trauma services. Geriatric patients with severe head injuries are less likely than their younger counterparts to be transferred to neurosurgical trauma centers. However, protocol-driven care of the elderly can reduce mortality due to head trauma through the application of the Brain Trauma Foundation guidelines [5]. National trauma policies need to be put in place to ensure that elderly patients receive the quality of care they rightly deserve.

## Consent

Written informed consent was obtained from the patient’s next of kin for publication of this case report. A copy of the written consent is available for review by the Editor-in-Chief of this journal.

## Abbreviations

CT: Computed tomography
GCS: Glasgow Coma Scale
TBI: Traumatic brain injury.
